# Compounding Plasmon–Exciton Strong Coupling System with Gold Nanofilm to Boost Rabi Splitting

**DOI:** 10.3390/nano9040564

**Published:** 2019-04-07

**Authors:** Tingting Song, Zhanxu Chen, Wenbo Zhang, Limin Lin, Yanjun Bao, Lin Wu, Zhang-Kai Zhou

**Affiliations:** 1 State Key Laboratory of Optoelectronic Materials and Technologies, School of Physics, Sun Yat-sen University, Guangzhou 510275, China; songtt6@mail2.sysu.edu.cn (T.S.); gsczx@gpnu.edu.cn (Z.C.); zhangwb29@mail2.sysu.edu.cn (W.Z.); linlimin0726@163.com (L.L.); baoyj3@mail.sysu.edu.cn (Y.B.); 2 School of Optoelectronic Engineering, Guang Dong Polytechnic Normal University, Guangzhou 510665, China; 3 Institute of High Performance Computing, A*STAR (Agency for Science, Technology and Research), 1 Fusionopolis Way, Connexis, Singapore 138632, Singapore; wul@ihpc.a-star.edu.sg

**Keywords:** plasmonics, nanoparticles, plasmon–exciton strong coupling, nanoscale building block

## Abstract

Various plasmonic nanocavities possessing an extremely small mode volume have been developed and applied successfully in the study of strong light-matter coupling. Driven by the desire of constructing quantum networks and other functional quantum devices, a growing trend of strong coupling research is to explore the possibility of fabricating simple strong coupling nanosystems as the building blocks to construct complex systems or devices. Herein, we investigate such a nanocube-exciton building block (i.e. AuNC@J-agg), which is fabricated by coating Au nanocubes with excitonic J-aggregate molecules. The extinction spectra of AuNC@J-agg assembly, as well as the dark field scattering spectra of the individual nanocube-exciton, exhibit Rabi splitting of 100–140 meV, which signifies strong plasmon–exciton coupling. We further demonstrate the feasibility of constructing a more complex system of AuNC@J-agg on Au film, which achieves a much stronger coupling, with Rabi splitting of 377 meV. This work provides a practical pathway of building complex systems from building blocks, which are simple strong coupling systems, which lays the foundation for exploring further fundamental studies or inventing novel quantum devices.

## 1. Introduction

Coherent states allow long-distance quantum information transportation and stable quantum information processing over long time durations [1,2,3]. Such coherent quantum states can be generated by tuning the interaction between quantum emitters and cavities into the strong coupling regimes. Recently, various strong coupling systems, consisting of quantum emitters and cavities, have been developed and successfully applied into the field of quantum physics [4,5], electronics [6], chemistry [7], and biology [8].

Generally, there are two types of cavities, i.e., dielectric optical microcavities (e.g., photonic crystal, microdisk, micropillar) [9,10] and plasmonic nanocavities (e.g., nanocube, nanorod, triangular nanoprism) [11,12,13]. As compared to microcavities, plasmonic cavities have two advantages. First, the plasmonic cavity is very famous for its ability to highly confine light within a sub-10 nm scale, leading to a mode volume (*V*_m_) smaller than 100 nm^3^ [14,15]. Recently, even a picocavity, which can localize light to volumes well below 1 nm^3^, has been proposed in a plasmonic system [16]. Since the coupling strength g is in inverse ratio to *V*_m_, strong couplings with large Rabi splitting Ω (Ω = 2g) have been widely enabled [17,18,19,20,21,22] by plasmonic cavities. Additionally, the ultrafast Rabi oscillations [23], strong coupling at the single-exciton level at room temperature [14,15,24], and ultrastrong coupling [25,26,27] have also been reported in plasmonic cavity systems.

Second, the nanometer-scale sizes of plasmonic nanocavities allow compact on-chip integration with a small footprint. They are, in fact, ideal nanoscale building blocks (i.e., elementary units of construction or composition) for constructing complex systems or functional devices. In fact, extensive research efforts have been devoted into the investigations of employing bare plasmonic nanostructures (such as nanorod, nanowire, etc.) as basic building blocks to constitute complex and functional systems [28,29,30], triggering out numbers of important optical applications [31,32,33]. Nevertheless, the feasibility for plasmon–exciton strong coupling systems working as building blocks to construct new systems is seldom explored. Driven by the trend of establishing quantum chips or quantum networks, it is highly desirable to design and study simple plasmon–exciton strong coupling systems and to study how to use them as the building blocks to construct complex strong coupling systems.

In this work, we demonstrate such a simple plasmon–exciton strong coupling system, composed of an Au nanocube (AuNC) and excitonic J-aggregate molecules, i.e., an AuNC@J-agg system. Working as the building block, we transfer it onto an Au nanofilm (AuNF) to obtain a complex strong coupling system, i.e., AuNC@J-agg/AuNF system. This complex system exhibits ~2.5× wider Rabi splitting than the building block of an AuNC@J-agg system. The tremendous enhancement on the plasmon–exciton coupling strength marks the feasibility of the building block concept. Our findings provide a route for establishing complex strong coupling systems with simple ones as building blocks, which would stimulate more fundamental studies or more functional quantum-device designs.

## 2. Materials and Methods

### 2.1. Synthesis of AuNC

The AuNC nanoparticles were synthesized by a seeded growth method [34]. Gold seeds were prepared by the rapid reduction of Au salts with the presence 50 μL of HAuCl_4_ (10 mM), 1.5 mL of Cetyltrimethylammonium bromide (CTAB, 100 mM), and 160 μL of NaHB_4_ (100 mM). After rapidly stirring for a few minutes, the yellow solution became brown. The seed solution was kept at 28 °C for 3 h to decompose the redundant reductant. At last, the solution was diluted (1:10) in deionized water (DI water) to prepare the gold nanoparticles.

A growth solution was synthesized by adding 8 mL of DI water, 0.2 mL of HAuCl_4_ (100 mM), 1.6 mL of CTAB (100 mM, 200 mM, 300 mM), 950 μL of L-ascorbic acid (AA, 100–800 mM), and diluted Au seed solution (0.5–5 μL) in sequence. The shape and size of AuNCs depended on the ratio of CTAB to AA and the volume of the Au seeds, respectively. The growth solution was thoroughly mixed and kept in a bath at 32 °C for 15 min. The colorless solution became purple red. The resultant nanoparticles were centrifuged (7570 r min^−^^1^, 3 min) twice to remove excess reagents and were re-dispersed in water.

### 2.2. Synthesis of AuNC@J-agg and AuNC@J-agg/AuNF systems

We needed to prepare the J-aggregate before fabricating the AuNC@J-agg hybrid. Briefly, 2 mL of PIC monomer solution (0.5 mM) was kept at 85 °C for 10 min. After adding 588 mg NaCl, the solution was kept at 85 °C for 3–4 min to dissolve the aggregate completely in monomer solution. Finally, there was a cooling process from 85 to 23 °C, during which the J-aggregates were gradually formed, with the color changing from red to orange. Then, 3 mL of AuNC solution was re-dispersed in 400 μL DI water, followed by adding 80–100 μL of J-aggregates (0.5 mM), and reacted for 10 min at room temperature. After that, 1.6 mL of DI water was added and the solution was centrifuged (4000 r min^−1^, 10 min) twice, and re-dispersed in DI water. Additionally, an Au film of ~100 nm was deposited by electron beam evaporation (Wavetest DE400, Texas Instruments, Dallas, TX, America) for constructing the AuNC@J-agg/Au NF system.

### 2.3. Optical Characterizations and Numerical Simulations 

The extinction spectra were collected by a UV-VIS-NIR spectrometer (Lambda 950, PerkinElmer, Waltham, MA, America). The Scanning electron microscopy (SEM) images were taken by an Auriga-39-34 (Zeiss Inc., Oberkchen, Germany) microscope operating at 5.0 kV. The transmission electron microscopy (TEM) images were taken by a JEM-1400 TEM machine (JEOL Inc., Tokyo, Japan), operated at 120 kV. Dark field scattering measurements images and spectra were recorded by a dark field optical microscope (Olympus BX53M, Olympus Inc., Tokyo, Japan) and a spectrograph (SP2500, Princeton, NJ, America). The numerical simulation was calculated by the finite-difference time-domain (FDTD) method.

## 3. Results

**Figure 1:**
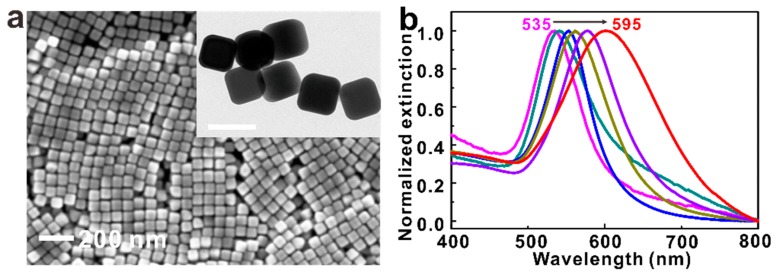
Synthesized Au nanocubes and their plasmonic properties. (**a**) The SEM and TEM images of the as-prepared AuNCs. The scale bar is 100 nm in the inset TEM image. (**b**) The extinction spectra of synthesized AuNCs, demonstrating tunable plasmon resonance mode from 535–595 nm.

We choose AuNCs in our experiments for two reasons. First, the NCs sustain large electric field enhancements and small mode volumes (typically at the corners and edges), which is beneficial for realizing strong coupling. Second, the high symmetry of the cube geometry makes it suitable for being building blocks. We examine the morphology of the as-prepared NCs using SEM and TEM, as presented in Figure 1a. Both SEM and TEM images confirm the uniformity of the shapes and sizes of our AuNCs, with an average side length of 85 nm. By changing the ratio of raw materials in the synthesis process, it is easy to vary the size of the AuNC in a controllable way, which leads to the tunablility of their plasmon resonance mode from 535–595 nm (Figure 1b). This wavelength range of plasmon resonance mode nicely covers the dipole transition frequency of J-aggregates (~580 nm), providing the possibility for strong coupling in the AuNC@J-agg hybrid.

**Figure 2:**
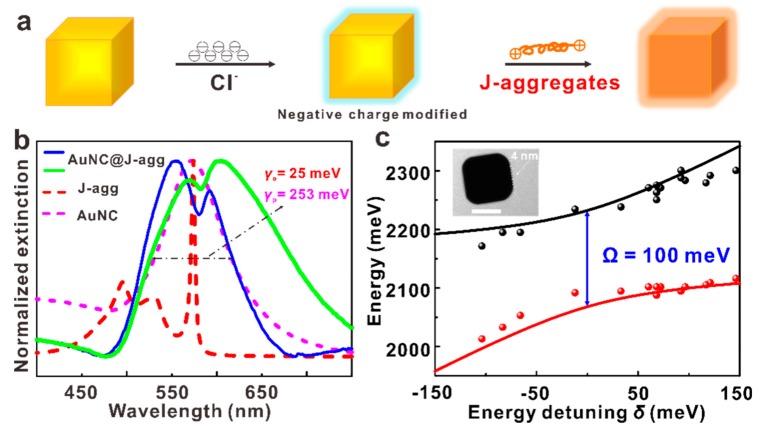
The AuNC@J-agg strong coupling systems. (**a**) Model diagram of preparation of AuNC@J-agg hybrid. (**b**) The extinction spectra of AuNC, J aggregates, and two representative AuNC@J-agg hybrids with different energy detuning. The decays of AuNC and J aggregates are 253 and 25 meV, respectively. (**c**) The anti-crossing curve of hybrid systems with a splitting energy of 100 meV. The inset picture is a typical TEM image of the AuNC@J-agg hybrid. The thickness of the J aggregate layer is 4 nm. The scale bar is 50 nm.

Aggregates are widely employed to construct a strong coupling system because J-aggregate is a type of molecule with large exciton momentum, which helps the realization of strong coupling [11,14,19]. Additionally, it is convenient to integrate J-aggregates with plasmonic nanocavities. As the J-aggregates hold positive charges, the AuNCs are firstly modified by negative charges (Cl^−^ ions). Upon mixing them together, the J-aggregates would be uniformly coated on the surface of AuNCs by electrostatic self-assembly (Figure 2a). As compared with the bare AuNCs, the extinction spectra of the coupled AuNC@J-agg hybrid splits into two peaks, with the central dip frequency around the transition frequency of the J-aggregates (Figure 2b). The two peaks stem from the two eigenstates of the coupled system, where the eigenvalues (ω_+_ and ω_−_) can be estimated from the measured frequency of these two peaks. Theoretically, according to the quantum mechanical Jaynes–Cummings picture, the two eigenvalues are given as follows [35]:

(1) $$\omega_{\pm }=\frac{1}{2}\left(\omega_{p}+\omega_{e}\right)\pm \sqrt{g^{2}+\frac{\delta^{2}}{4}},$$

where ω_p_ and ω_e_ are the resonance energies of the plasmonic cavity and exciton of J-aggregates, *g* is the coupling strength of plasmonic cavity and exciton, and δ = ω_p_ – ω_e_ is energy detuning. By plotting the measured (symbols) and calculated eigenvalues (solid lines) as the function of the energy detunings δ, in Figure 2c, we show a clear anticrossing feature, with excellent agreement between experiments and calculations. The Rabi splitting width Ω (Ω = 2g) is estimated to be 100 meV at zero energy detuning, which meets the strong coupling criterion of the AuNC@J-agg hybrid (i.e., Ω > [(γ^2^_p_ + γ^2^_e_)/8]^1/2^ [14], where γ_p_ and γ_e_ are the decay of plasmon cavity and exciton). The anticrossing curve, together with the large splitting width, clearly demonstrates the plasmon–exciton strong coupling in our AuNC@J-agg hybrid.

It is noted that the coupling strength is proportional to the number of coupling excitons [18,19], but there is always a saturation number for the coupling excitons in one single plasmonic cavity [14,35]. In our system, the number of molecule excitons can be adjusted by changing the thickness of J-aggregate layer and it is found that the excitons reach the saturation number when the thickness of the J-aggregate layer is about 4 nm, as a thicker layer hardly brings about wider Rabi splitting. Therefore, in our following experiments, the thickness of the J-aggregate is fixed at 4 nm (as shown by the inset of Figure 2c).

**Figure 3:**
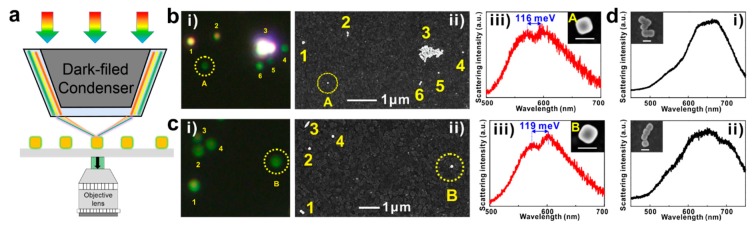
Characterization of a single AuNC@J-agg hybrid. (**a**) The model diagram of dark field microscopy. (**b**) and (**c**) The results of dark field measurements for two individual AuNC@J-agg hybrids. **i**) Dark field image. **ii**) Corresponding SEM image. **iii**) The dark field scattering spectra of points A and B, which correspond to two individual AuNC@J-agg hybrids displaying Rabi splitting of 116 meV and 119 meV. Inset: SEM images of single AuNC@J-agg hybrids. (**d**) The dark field scattering spectra of close-packed AuNC@J-agg hybrids, which cannot exhibit Rabi splitting. Inset: SEM images of assembling AuNC@J-agg hybrids. The scale bars are 100 nm.

To deepen the understanding of the strong coupling properties of the AuNC@J-agg hybrid, as well as the optical behaviors of more complex strong coupling systems, a dark field measurement system was developed to collect the scattering signals of a single AuNC@J-agg hybrid (Figure 3a). Figure 3b,c illustrate the typical dark field measurement results of two individual AuNC@J-agg hybrids on the ITO substrate.

To be more specific, the individual AuNC@J-agg hybrids were dispersed on the ITO substrate and these hybrids were firstly measured by dark field microscopy. Figure 3bi),ci) are the dark field images, where those light points (such as the points A and B) represent the AuNC@J-agg hybrids. Following the finding of these light points, we collected their scattering signals. After that, the ITO substrate (with individual AuNC@J-agg hybrids on its surface) were transferred to a SEM to obtain high magnification images. During the observation of SEM measurement, we particularly searched the area where we collected the scattering signals, such as Figure 3bii),cii), which are the SEM images of the area around points A and B. Lastly, by comparing the special patterns formed by different points in both the dark field images and the SEM images (like the points 1–6 in both Figure 3bi),ii)), we are able to match the each light point in the dark field image with its corresponding SEM image. Therefore, we can match the obtained dark field scattering spectrum with its corresponding AuNC@J-agg individual, and the scattering spectrum and SEM image for points A and B are presented in Figure 3biii),ciii), respectively. These dark field measurements have been extensively used in the study of single nanoparticle systems (including our group) [14,21,22,36,37,38], demonstrating high reliability and stability. In our dark field measurements, close-packed AuNC@J-agg hybrids were also collected (Figure 3d). However, the assembling of AuNC can lead to a large plasmon mode shift [36], making it so the close-packed AuNC systems cannot match the dipole transition energy of J-aggregates exciton and fail to generate strong coupling due to large energy detuning (no Rabi splitting). So, in the following experiments, we only focus our exploration on individual AuNC@J-agg hybrids.

**Figure 4:**
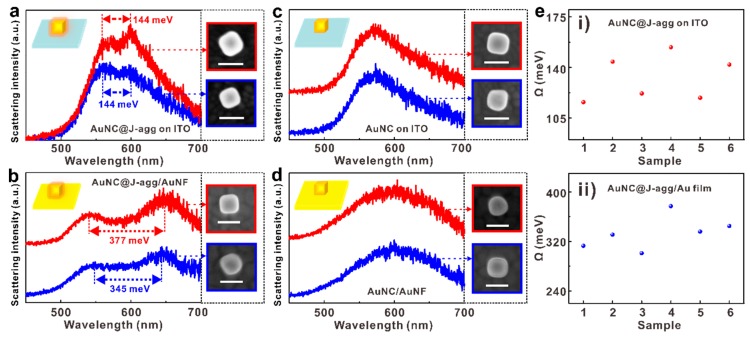
Characterization of complex strong coupling systems. (**a**) Scattering spectra of two AuNC@J-agg hybrid samples on the ITO substrate, with Rabi splitting of 144 meV. (**b**) Scattering spectra of two AuNC@J-agg/AuNF samples, with Rabi splitting of 327 meV and 341 meV. (**c**) Scattering spectra of two AuNC samples on the ITO substrate. (**d**) Scattering spectra of two AuNC on AuNF samples. (**e**) The comparison of Rabi splitting energy of AuNC@J-agg on the ITO and AuNC@J-agg/AuNF systems, which are statistically collected from 12 samples.

With this reliable dark field microscopy technique to characterize single nanoparticle systems, we are now ready to compare the optical behaviors of different systems, e.g., the simple building block AuNC@J-agg hybrids on the ITO substrate (Figure 4a), the complex AuNC@J-agg/AuNF system (Figure 4b), the simple plasmonic AuNC on the ITO substrate (Figure 4c), and the complex AuNC/AuNF plasmonic system (Figure 4d). In doing so, the scattering spectra of two samples for each system are presented in red and blue curves, with the SEM images of their corresponding individuals marked by red and blue squares placed on the right side of the spectra, respectively.

Comparing Figure 4a,b, the splitting width is measured to be about 144 meV when the AuNC@J-agg hybrid is placed on the ITO substrate. On the contrary, when the AuNC@J-agg hybrid is put on the surface of an Au nanofilm, forming the AuNC@J-agg/AuNF complex system, the splitting width increases to 345–377 meV, which is about ~2.5× enhancement, as compared to that of the AuNC@J-agg hybrid building block. We also provide the statistic results of splitting width from different samples for both the AuNC@J-agg hybrid and the AuNC@J-agg/AuNF complex system in Figure 4e, which evidently confirms the wider splitting and stronger coupling in the complex system.

To exclude the possibility that the splitting in scattering spectra is caused by the plasmon mode coupling of AuNC and AuNF, the scattering spectra of pure plasmonic AuNC on the ITO substrate or the AuNF substrate have also been shown in Figure 4c,d. Clearly, there is only one scattering peak for all the samples of these two systems. Additionally, based on the scattering spectra and the strong coupling criterion [14], the strong coupling threshold is calculated as Ω = 143 meV for the complex AuNC@J-agg/AuNF system. Therefore, the large Rabi splitting of over 300 meV indeed indicates a plasmon–exciton strong coupling in the AuNC@J-agg/AuNF system. Note that there are some previous works about the study of plasmon–exciton strong coupling based on systems including nanoparticles on AuNFs and excitonic molecules [35,39,40], where the molecules are fabricated into thin molecular layers attached on the surface of AuNFs. Different from these studies, in our investigations, the excitonic J-aggregates were firstly coated around AuNC, generating a plasmon–exciton strong coupling system (i.e., the AuNC@J-agg hybrid). Then, in order to explore the possibility of constructing a new strong coupling system with a simple one, we transferred the AuNC@J-agg hybrid onto AuNF, constructing a new strong coupling system, which is the AuNC@J-agg/AuNF complex system.

**Figure 5:**
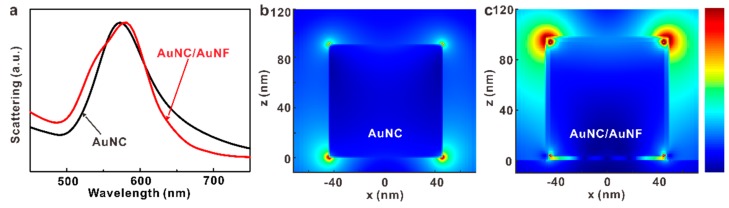
Numerical simulations. (**a**) The calculated scattering spectra of an AuNC (black curve) and AuNC with a 4 nm thick dielectric coating layer on the AuNF substrate (red curve). The side length of AuNC is 90 nm and the refractive index of the dielectric coating layer is set at 1.6. (**b**) and (**c**) Electric field distributions of the structures of (**b**) pure AuNC and (**c**) AuNC on AuNF.

Numerical simulations (Figure 5) can also help in explaining the differences between the complex AuNC@J-agg/AuNF system and the simple AuNC@J-agg system. Using the FDTD method, we analyze the plasmonic modes and electric field distributions of a single AuNC and a single AuNC on Au nanofilm (AuNC/AuNF). In order to simulate the AuNC@J-agg/AuNF system, a 4 nm thick dielectric layer, with reflective index of 1.6, is included around the AuNC (side length of 90 nm), when it is placed on the Au film. The permittivity of Au is taken from Johnson and Christy [41].

From Figure 5a, we observe plasmon resonant modes with similar resonance energies (peak at 570 or 580 nm) for both structures of the individual AuNC and AuNC/AuNF, which provides the possibility that excitons of J-aggregates can strong couple with the plasmon modes of these two structures. In other words, if a large spectral shift happens after the integration of strong coupling building blocks, the new system can hardly achieve strong coupling due to the energy detuning. The resonant mode of the AuNC/AuNF structure only has a small redshift, as compared to the bare AuNC, which is in good agreement with a previous report [42]. The electric field distributions of the two structures, at their resonant wavelengths, are compared in Figure 5b,c. It is obvious that the AuNC/AuNF shows stronger electric field enhancements than the pure AuNC, especially at the two upper corners. As the coupling strength g is proportional to the electric field enhancements [1,43], it is reasonable to obtain the much larger Rabi splitting in the complex system of AuNC@J-agg/AuNF.

Since the structure of the nanoparticle on AuNF is widely applied for studying strong coupling, two main differences in our paper should be mentioned. First, for most previous studies, it is the gap plasmon mode that participates in the strong coupling, while our study it is not. According to previous studies, it is generally known that the gap plasmon mode can concentrate light entirely in the gap between the nanoparticle and the AuNF, nearly without electric field enhancements at the upper side of the nanoparticles [15,39]. However, Figure 5c shows that the strongest electric field enhancements are around upper corners of the AuNC, vividly demonstrating that the plasmon mode applied in our study is a normal hybridization mode of the AuNC/AuNF structure, not the gap plasmon mode. We also want the gap plasmon mode to be applied in our study, because it sustains extremely strong electric field enhancements. However, calculations indicate that the gap plasmon of our AuNC/AuNF structure is at ~1160 nm, which is quite far away from the transition energy of J-aggregates (~ 580), making it can hardly strong couple with J-aggregates. Second, the upper side of AuNC exhibits strong electric field enhancements and the J-aggregates are coated around the AuNC, so the strong coupling in our AuNC@J-agg/AuNF system also involves J-aggregates on the upper side. In addition, since the electric field enhancement around the upper side is more intense than that in the gap, it is reasonable to believe that the J-aggregates of the upper side play important roles for the Rabi splitting enhancement.

## 4. Conclusions

In conclusion, we have achieved strong coupling in a simple system of AuNC@J-agg, which is fabricated by coating AuNCs with J-aggregate molecules. The extinction spectra of AuNC@J-agg assembles, the anticrossing relation of the two eigenvalues dependence on energy detuning of AuNC, and the exciton of J-aggregates, as well as the scattering spectra of single AuNC@J-agg hybrids, evidently demonstrate the strong plasmon–exciton interaction of the AuNC@J-agg hybrid. Additionally, we prove that the simple strong coupling system of the AuNC@J-agg hybrid can serve as a building block to construct more complex strong coupling systems, taking the AuNC@J-agg/AuNF system as an example. Dark field measurements of various single particle systems show that the AuNC@J-agg/AuNF systems exhibit Rabi splitting ~2.5× larger than that in the AuNC@J-agg hybrid, which indicates stronger plasmon–exciton coupling. Moreover, numerical calculations indicate that the stronger coupling can be attributed to the larger electric enhancements in the complex system of AuNC@J-agg/AuNF. This work has successfully proved a useful building block concept, where simple plasmon–exciton strong coupling systems can be exploited as the building blocks to construct complex systems and to obtain stronger light-matter coupling. This exhibits great potential in quantum technologies, e.g., building quantum networks or other quantum devices based on strong coupling.
